# Tetraspanin8 expression predicts an increased metastatic risk and is associated with cancer-related death in human cutaneous melanoma

**DOI:** 10.1186/s12943-021-01429-0

**Published:** 2021-10-02

**Authors:** Odile Berthier-Vergnes, Laetitia Barbollat-Boutrand, Roxane M. Pommier, Arnaud de la Fouchardière, Patrick Combemale, Maxime Grimont, Noémie Lopez-Ramirez, Julie Caramel, Stéphane Dalle, Jean-Luc Perrot, Caroline Gaudy-Marqueste, Nicolas Macagno, Sandrine Mansard, Fanny Bouquet, Ingrid Masse

**Affiliations:** 1 CNRS, UMR5534, Centre de Génétique et de Physiologie Moléculaires et Cellulaires, F-69622 Villeurbanne, France; 2 Centre de Recherche en Cancérologie de Lyon, UMR Inserm 1052 CNRS 5286 Centre Leon Berard, Batiment Cheney D, 2eme etage, 28 rue Laennec, 69373 Cedex 08 Lyon, France; 3 Département de Biopathologie, Centre Leon Bérard, Lyon, France; 4 Department of Dermatology, University Hospital of St-Etienne, Saint-Etienne, France; 5 Dermatology and Skin Cancer Department, Aix-Marseille University, 264, rue Saint-Pierre, 13385 Marseille, France; 6 Department of Dermatology, University Hospital Estaing, Clermont Ferrand, France; 7 Institut Roche, Boulogne-Billancourt, France

Cutaneous malignant melanoma remains an aggressive cancer given its high metastatic proclivity. Numerous patients with newly diagnosed cutaneous melanoma have early lesions, which can be cured by surgery. When patients have to face advanced-stage melanomas with distant metastases, the mean survival time remains low, despite the development of recent therapies [1]. The conventional diagnostic and prognostic biomarkers (i.e. Clark Level (CL), Breslow Index (BI) or immunohistochemical markers [2]) seem insufficient to distinguish precisely primary epidermis-limited neoplasms from aggressive or advanced/metastatic melanomas, and to assess the outcome for individual patients. Thus, additional relevant biomarkers are needed to predict the individual risk of metastasis and monitor disease progression.

We previously showed that Tspan8, a known inducer of invasion in carcinomas, is sufficient to confer invasiveness to non-invasive melanoma cells [3–5]. We demonstrated that Tspan8 expression is sufficient to confer invasive properties to non-invasive melanoma cells in boyden assays in 2D-cultured cells [3, 4] but also in 3D skin-reconstructs models [5]. Moreover, we identified several Tspan8 transcriptional regulators whose deregulation leads to Tspan8 expression [3, 5, 6], which could help to design new therapeutic strategies targeting Tspan8. Indeed, Tspan8 emerges recently as a blood biomarker [7] but also as a promising therapeutic target in various carcinomas, since Tspan8 blocking antibodies decreased angiogenesis [8], cell motility [9], tumor growth in mice [10] and metastasis [11, 12]. We showed that early melanoma spreading in skin-reconstruct models was reduced by Tspan8-specific antibody [5]. Tspan8 could also be a relevant target for radio-immunotherapy [12]. In this context, we wondered whether Tspan8, whose expression is highly correlated with acquisition of invasiveness, could be a potential new biomarker for early detection prognosis which could help to predict metastasis risk in individual patients, including those bearing thin melanomas that, in some cases, can metastasize and cause patients deaths.

Here, we demonstrated that Tspan8 was more frequently expressed in metastatic samples and that Tspan8 expression was correlated with the presence of a BRAF^V600E^ mutation, a higher propensity to give rise to distant metastases and an increased risk of death.

## Results and discussion

### High *TSPAN8* mRNA expression is linked to metastatic dissemination in the TCGA cohort

We previously showed that Tspan8 expression is restricted to invasive melanoma cell lines [13]. However, when Tspan8 expression was studied in the sixty-three cell lines from the Cancer Cell Line Encyclopedia (CCLE), very low levels of *TSPAN8* mRNA and protein were observed (sup. Figure 1a-c). Heterogeneous but easily detectable Tspan8 expression levels were observed in vitro only in short-term cultures derived from metastatic pleural effusions from patients developing a primary invasive melanoma [14] (unpublished results; sup. Figure 1d). We can surmise that classic establishment of cell lines, that favors cell proliferative capacities rather than invasiveness, leads to a low Tspan8 expression, which is not representative of Tspan8 expression observed in situ in primary melanoma samples [13]. To overcome this in vitro problem, we performed our studies in patient samples.

**Fig. 1:**
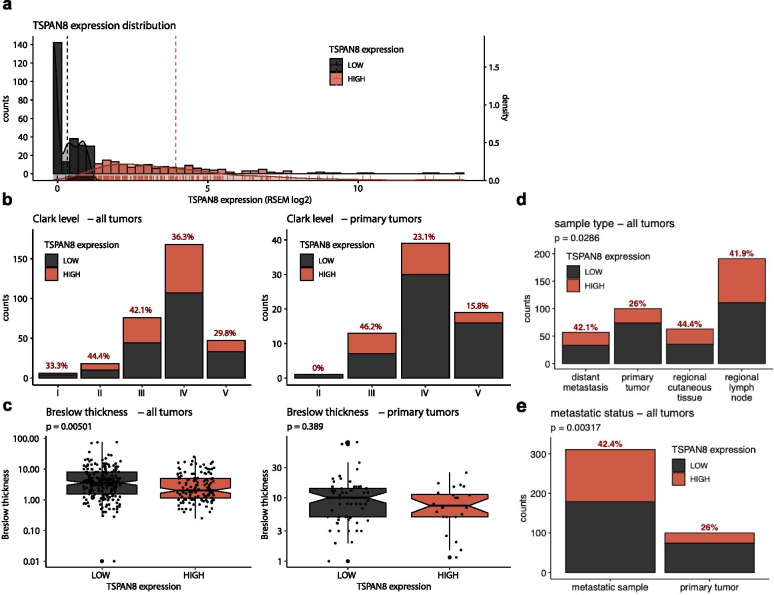
TCGA analysis shows that TSPAN8 mRNA expression correlates with metastases dissemination and reduced disease free survival of patients harboring cutaneous melanomas*.* **a** *TSPAN8* mRNA expression distribution across TCGA melanoma cohort: Gaussian finite mixture models (mclust R package) was applied and identified two Gaussian subdistribution in melanoma (TCGA RSEM log2), one relying to absent/low *TSPAN8* expression tumors (black lines) and the other to high *TSPAN8* expression tumors (red lines). Each bar on the x axis corresponds to one tumor. **b** Tumor number according to Clark’s level versus *TSPAN8* mRNA expression: for each level of Clark classification, the number of tumors expressing absent/low or high TSPAN8 mRNA as well as the percentage of TSPAN8+ tumor are presented for all TCGA samples (*n* = 414; left panel) or for primary tumors only (*n* = 100; right panel). **c,** Sample distribution according to their Breslow thickness for tumors with absent/low or high *TSPAN8* expression levels, in the totality of TCGA samples (n = 414; left panel) or for primary tumors only (n = 100; right panel). Wilcoxon rank-sum tests. **d** Tumor number according to the type of samples (primary tumor, regional cutaneous tissue, regional lymph node or distant metastasis): for each category, the number of tumors expressing absent/low or high TSPAN8 mRNA as well as the percentage of TSPAN8+ tumor is presented for all TCGA samples (n = 414). Fisher’s exact test. **e,** Number and percentage of samples expressing TSPAN8 mRNA at high level versus low (or absent) level according to the location: in primary tumors or in metastatic samples (n = 414). Fisher’s exact test

We first analyzed *TSPAN8* mRNA expression in the data set from The Cancer Genome Atlas (TCGA; https://cancergenome.nih.gov/) and observed a heterogeneous expression. To decipher the inner structure of the *TSPAN8* expression distribution across the cohort, we applied Gaussian finite mixture models (mclust R package) and highlighted 2 groups of tumors: 1/3 of samples showed absent/low (in black) *TSPAN8* mRNA expression and 2/3 of samples displayed a higher one (in red) (Fig. 1a). These two groups have been used for further analyses.

No correlation was found with CL, neither on the totality of the 414 samples nor on the 100 primary samples only (Fig. 1b). However, BI analysis surprisingly revealed that melanoma samples displaying a high *TSPAN8* expression were more frequently thin melanomas (Fig. 1c, left panel). This highly significant effect was completely lost when only primary melanomas were analyzed (Fig. 1c, right panel), that can be explain by the fact that, in the TCGA cohort, mRNA expression data from primary melanomas are available only for thick melanomas (sup. Figure 2a), probably due to the small amount of tumor material for thin melanomas. Nevertheless, in this cohort, all thin melanomas gave rise to metastases, which could link *TSPAN8* mRNA expression to metastatic properties.

**Fig. 2:**
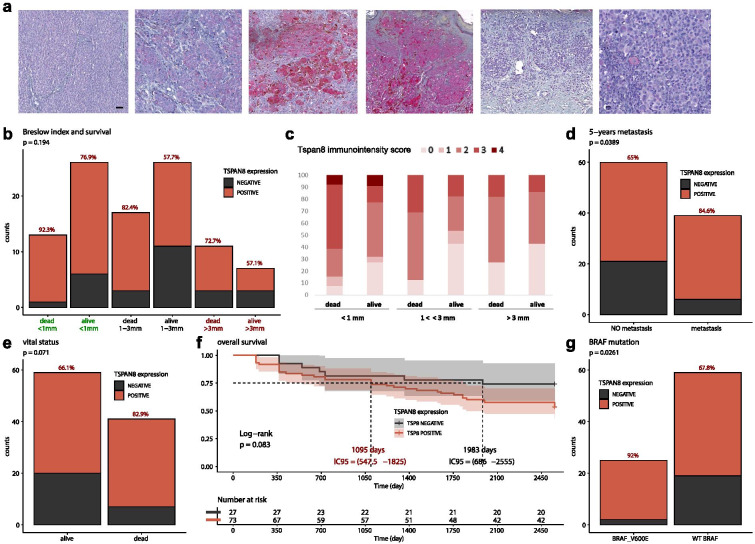
Tspan8 protein expression can be immunoscored in primary melanomas and correlates with a higher propensity to give rise to distant metastases, an increased risk of death and the presence of a BRAF^V600E^ mutation*.* **a** Tspan8 expression detected by immunochemistry in different melanoma samples from immunoscore 1 to 4 (4 first pictures). **b,** Repartition of primary melanomas according to the Breslow Index (inferior to 1 mm, between 1 and 3 mm, superior to 3 mm), the patient status 5 years after diagnosis (dead or alive) and Tspan8 protein expression (positive or negative): for each category, the percentage of Tspan8+ tumors are presented (n = 100). Fisher’s exact test. **c** Tspan8 immunostaining classified in 5 different immunoscores from no Tspan8 staining (score 0) to strong Tspan8 staining (score 4), according to the Breslow Index (inferior to 1 mm, between 1 and 3 mm, superior to 3 mm) and the patient status 5 years after diagnosis (dead or alive; n = 100). **d** Tumor number according to metastasis presence versus TSPAN8 protein expression: for patients developing or not metastases in the 5 years after diagnosis of their primary melanoma, the number of samples expressing or not Tspan8 protein as well as the percentage of Tspan8+ tumors are presented (n = 100). Fisher’s exact test. **e** Vital status of the patients according to Tspan8 protein expression: the number of samples expressing or not Tspan8 protein as well as the percentage of Tspan8+ tumors are presented (n = 100). Fisher’s exact test. **f** 5-years overall survival (n = 100). Log-rank test. **g** The repartition of tumors according to BRAF status and Tspan8 protein expression is presented. Fisher’s exact test

Accordingly, high TSPAN8 expression was observed in 44.4% of regional cutaneous tissues, 41.9% of regional lymph nodes and 42.1% of distant metastases, versus 26% for primary tumors (Fig. 1d). Overall, we observed that only 26% of primary melanomas displayed high *TSPAN8* expression, whereas local or distant melanoma dissemination was significantly associated with a more frequent TSPAN8 expression (Fig. 1e), consistently with the correlation of Tspan8 expression with invasiveness previously demonstrated in vitro [3, 4, 6, 13].

### Tspan8 protein expression in human primary melanomas was correlated with a higher propensity to give rise to distant metastases and an increased risk of death

To evaluate the impact of Tspan8 expression at the protein level, we performed Tspan8 immunohistochemical staining analysis in a cohort of 100 primary melanoma samples: 73% of primary melanomas were positive for Tspan8 protein expression (sup. Figure 2b), without any significant differences depending on patient age or gender (sup. Figure 2c,d). We analyzed Tspan8 immunostaining in details, and sections were scored positive if any reactive areas were seen in melanoma lesions with growing intensity from 0 (no staining) to 4 (strong staining) (Fig. 2a, four first panel). Tspan8 staining is often clonal with some positive clones neighboring some negative areas (Fig. 2a, third panel), but could also be detected in the invasive front of the tumor (Fig. 2a, fifth panel) and, even if the majority of melanoma cells harbored a cytoplasmic staining, some rare cells in some samples presented a stronger staining at the membrane (Fig. 2a, sixth panel).

In accordance with the TCGA cohort, no correlation with CL (sup. Figure 2e) or BI emerged (sup. Figure 2f,g). However, Tspan8 was more frequently expressed in samples from patients who died during the 5-year period after the melanoma diagnosis than in samples from patients who remained alive, and especially for thin melanomas, inferior to 1 mm (Fig. 2b). Tspan8 protein seemed more frequently expressed in the thinnest melanomas from patients dead during the 5-year period after diagnosis (92.3% of samples). Moreover, the thinnest primary melanomas seemed to express the highest levels of Tspan8 protein (immunointensity score 4 exclusively in < 1 mm samples), especially in patients’ dead 5-years after diagnosis (60% of immunointensity scores 3–4; Fig. 2c).

Besides, only 65% of primary tumors that did not give metastases expressed Tspan8 whereas in primary tumors that disseminated, 84.6% expressed Tspan8 (Fig. 2d), demonstrating that Tspan8 expression in primary tumors was significantly correlated with the propensity to metastasize. Interestingly, in thin melanomas (less than 1 mm) especially, 11 among 12 samples (91.7%) from patients who died during the 5 years after the melanoma diagnosis gave rise to metastasis and 10 of them (90.9%) expressed Tspan8 protein, strengthening the correlation between Tspan8 protein expression and metastatic dissemination in thin melanomas. These results are particularly interesting since it is known that patients bearing thinner melanomas with no deep cutaneous invasion have generally a better survival rate [15] but that some thin melanomas (< 0.75 – 1 mm) could acquire some aggressive properties and lead to patient death. Indeed, metastases in sentinel lymph node were detected in more than 5% of patients who had a sentinel lymph node biopsy after the detection of a thin melanoma (< 1 mm) [16]. Our data showed that Tspan8 immunostaining could thus be a biomarker to assess the individual risk of metastasis, especially for thin melanomas, and potentially linked with patient poor survival since Tspan8 expression seemed also correlated with the vital status of the patients. The proportion of Tspan8-expressing primary melanomas increased from 66.1% for alive patients to 82.9% for dead patients, 5 years after the diagnosis (Fig. 2e), and the 5-years overall survival curve (Fig. 2f) showed that the presence of Tspan8 protein in primary melanomas tended to represent an increase risk of death.

### Tspan8 expression was correlated with the presence of a BRAF mutation in patient melanoma samples

In melanoma cell lines in vitro, we were not able to highlight a possible correlation between *TSPAN8* mRNA expression and the presence of a BRAF or a NRAS mutation (sup. Fig. 3a), that are the most frequent genetic alterations detected in melanoma [17]. In the TCGA cohort as well, no statistical difference could be observed, neither for BRAF nor NRAS mutation (sup. Fig. 3b,c), but in our cohort of primary melanomas sample, a systematic screening of BRAF^V600E^ mutation by immunohistochemistry (sup. Fig. 3d) revealed a significant correlation between Tspan8 protein expression and the presence of the BRAF^V6OOE^ mutation (Fig. 2g). These data emphasize the functional interaction between Tspan8 and BRAF that we previously showed in vitro, suggesting that Tspan8 is a downstream effector of the RAF/MEK/ERK signaling pathway [3].

## Conclusion

Overall, our findings suggest that Tspan8 protein detection could help the early identification of subgroups of patients bearing primary melanomas, preferentially BRAF-mutated, with high risk of developing metastatic disease and could possibly discriminate thin melanomas with poor outcome. Although further investigations in a larger patient cohort are needed, these findings could have potential clinical relevance since Tspan8 protein expression can be used to define subgroups of thin melanoma patients who have a worse prognosis. Moreover, they should have important implications for proposed populations screening programs and patients’ cure. Indeed, since one third (4/12) of patients with a thin melanoma that metastasized and led to the patient death have a Tspan8 staining associated with a *BRAF*^*V600E*^ mutation, an anti-BRAF/MEK therapeutic strategy could be proposed.

## Supplementary Information

**Additional file 1: Supplementary Figure 1**. Tspan8 expression is detected in aggressive human melanoma cell lines. **Supplementary Figure 2**. Tspan8 expression analysis in TCGA cohort and a cohort of 100 human primary melanomas from archives of four French clinical centers. **Supplementary Figure 3**. TSPAN8 protein expression correlates with the presence of BRAFV600E mutation in primary melanomas. **Supplementary Methods.**

## Data Availability

The dataset used during this study are available from TCGA. All other data generated or analyzed during this study are included in this published article or are available from the corresponding author on reasonable request.

## Abbreviations

BI: Breslow Index
CCLE: Cancer Cell Line Encyclopedia
CL: Clark Level
KIRREL: Kin of IRRE-like protein 1
PCR: Polymerase Chain Reaction
TCGA: The Cancer Genome Atlas
TSPAN8: Tetraspanin 8
