# Supplementary material for: Tetraspanin8 expression predicts an increased metastatic risk and is associated with cancer-related death in human cutaneous melanoma
Source: Mol Cancer. 2021 Oct 2;20:127. doi: 10.1186/s12943-021-01429-0 (PMC8487126; doi:10.1186/s12943-021-01429-0)
Supplement: Supplementary file 1 — Additional file 1: Supplementary Figure 1. Tspan8 expression is detected in aggressive human melanoma cell lines. Supplementary Figure 2. Tspan8 expression analysis in TCGA cohort and a cohort of 100 human primary melanomas from archives of four French clinical centers. Supplementary Figure 3. TSPAN8 protein expression correlates with the presence of BRAFV600E mutation in primary melanomas. Supplementary Methods. [file 12943_2021_1429_MOESM1_ESM.docx]

**Supplementary data**

**Supplementary Figure 1**


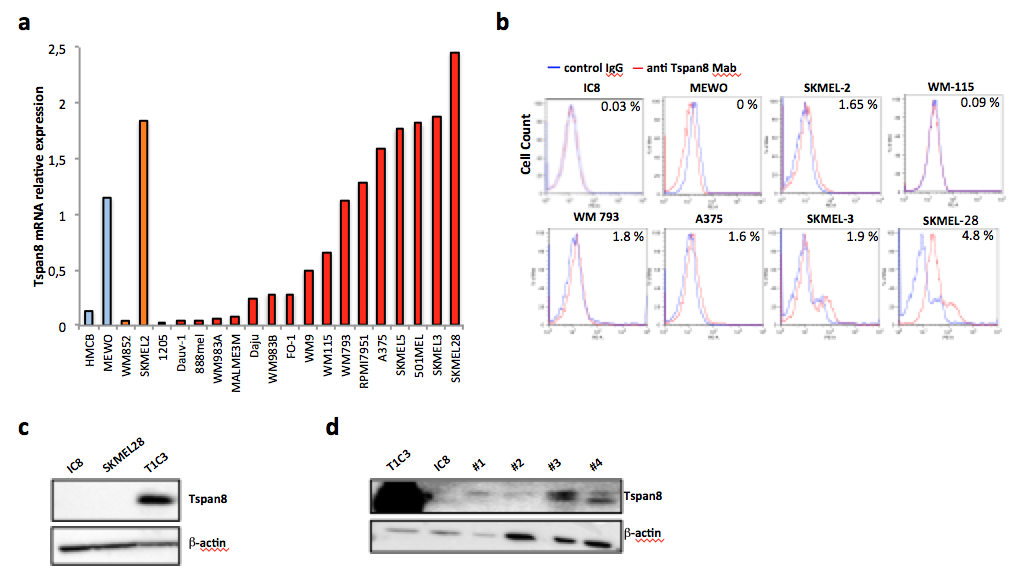


**Tspan8 expression is detected in aggressive human melanoma cell lines*.* a,** Tspan8 mRNA expression level measured by QPCR in 21 cell lines of the CCLE. **b,** Tspan8 protein expression monitored at cell surface by FACS analysis in 7 different cell lines, compared to the IC8 cell line that serves as a non-invasive negative control. **c,** Tspan8 protein expression detected by western blot in SKMEL28 cell line, compared to the IC8 and T1C3 cell lines, which serve as negative and positive controls respectively. **d,** Tspan8 protein expression detected by western blot in positive T1C3 and negative IC8 cell lines, as well as in 4 short-term cultures of metastatic pleural effusions from patients developing a primary invasive melanoma.

**Supplementary Figure 2**

**Tspan8 expression analysis in TCGA cohort and a cohort of 100 human primary melanomas from archives of four French clinical centers.** **a,** Repartition of the primary samples versus metastatic ones according to their Breslow thickness. **b,** Distribution of primary cutaneous melanomas according to the presence or absence of Tspan8 expression by immunohistochemistry staining (n=100). **c,** Repartition of patient age at diagnosis according to the presence or absence of Tspan8 expression (n=100). Wilcoxon rank-sum test. **d,** Tumor number according to the sex of the patients: for females and males, the number of tumors expressing or not Tspan8 protein as well as the percentage of TSPAN8+ tumor are presented for primary tumors (n=100). Fisher’s exact test. **e,** Tumor number according to Clark level versus TSPAN8 protein expression: for each class of Clark level, the number of primary melanomas expressing or not Tspan8 protein as well as the percentage of Tspan8+ tumor are presented (n=100). Fisher’s exact test. **f,** Breslow thickness according to the absence or presence of Tspan8 protein expression (n=100). Wilcoxon rank-sum test. **g,** Tumor number according to Breslow thickness versus TSPAN8 protein expression: for each class of Breslow Index, the number of primary melanomas expressing or not Tspan8 protein as well as the percentage of Tspan8+ tumors are presented (n=100). Fisher’s exact test.

**Supplementary Figure 3**

**
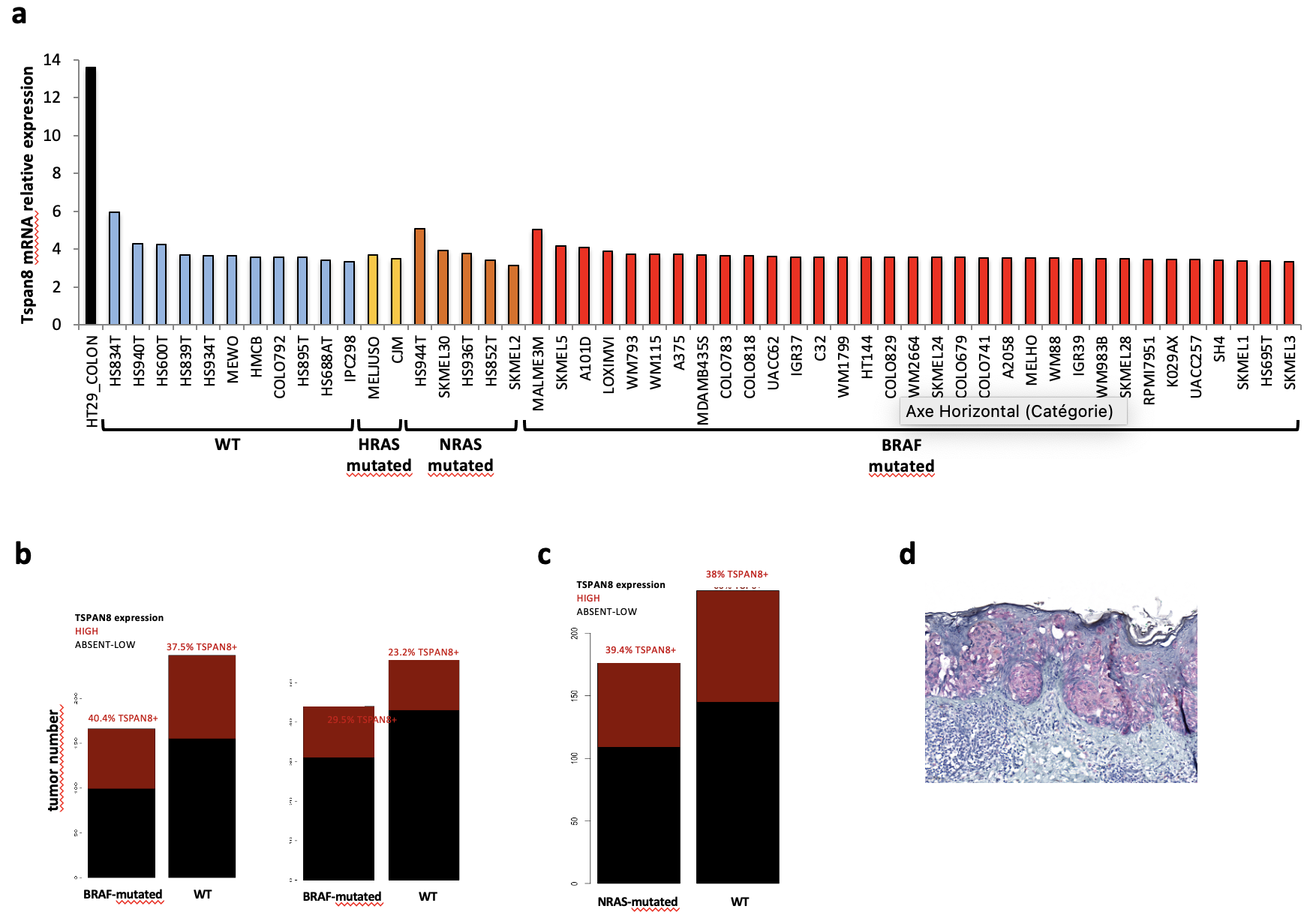
TSPAN8 protein expression correlates with the presence of BRAFV600E mutation in primary melanomas.** **a,** Tspan8 mRNA expression in 63 cell lines from the CCLE according to the status of HRAS, NRAS and BRAF mutations. **b,** TSPAN8 mRNA expression according to BRAF status for all TCGA samples (n=414; left panel) or for primary tumors only (n=100; right panel). **c,** TSPAN8 mRNA expression according to NRAS status for all TCGA samples (n=414). **d,** Immunostaining with a mouse monoclonal antibody detecting BRAFV600E mutation in a melanoma sample.

**Supplementary Methods**

*Cell lines and cell culture*

HMCB, MEWO, WM 852, SKMEL2, 1205, Duv-1, 888mel, MALME3M, Daju, WM983B, FO-1, WM9, WM115, WM793, RPMI7951, A375, SKMEL5, 501MEL, SKMEL3 and SKMEL28 cell lines were cultured as monolayers in DMEM or McCoy’s 5A medium (Gibco, Paisley, UK) supplemented with 10% fetal calf serum, 100 IU/ml penicillin and 100 IU/ml streptomycin. Short-term cultures were established from pleural effusion of different patients developing aggressive melanomas and cultured as previously described (Ohanna et al, Genes Dev, 2018). Cultures were regularly screened for mycoplasma contamination using the Hoechst 33258 fluorescence staining procedure.

*Protein extraction and western blotting*

Total proteins were extracted exactly as previously described (Agaësse et al, Oncogene, 2017). Tspan8 was detected using a mouse monoclonal anti-Tspan8 antibody (TS29 clone 1/2000; Le Naour et al, Mol Cell Proteomics, 2006). As a loading control, β-actin was detected with a mouse monoclonal anti-Actin Clone C4 antibody (1/5000; Millipore, Darmstadt, Germany). Western blot quantifications were performed using ImageJ software : (ImageJ, RRID:SCR_003070).

*Real-time RT-qPCR*

Total RNA was extracted from cell line cultures using the RNAeasy mini-kit (Qiagen, Germantown, MD, USA), reverse-transcribed into cDNA using the PrimeScript RT Reagent kit (Takara, Shiga, Japan) and analyzed by real-time qPCR using SYBR Premix ExTaqII (Takara, Shiga, Japan) on a Mx3000P real-tim PCR system (Stratagene, Santa Clara, CA, USA). Results were normalized to the 18S rRNA expression level. The primers used are previously described (Agaësse et al, Oncogene, 2017).

*TCGA melanoma data set analysis*

Data from 414 melanoma samples generated by the TCGA Research Network (<http://cancergenome.nih.gov/>; (The Cancer Genome Atlas, RRID:SCR_003193)) were used for analysis of *TSPAN8* mRNA expression. Clinical, mutation and mRNA expression data (RNASeq V2 RSEM) were extracted from cbioportal (<https://www.cbioportal.org/>) (Cerami et al, Cancer Discovery, 2012; Gao et al, Science Signaling, 2013). The original cohort encompassed 478 patients that we filtered out by the unambiguous status of primary melanoma diagnosis and TSPAN8 expression availability. Duplicated patients were also filtered out.

*CCLE data set analyses*

The levels of mRNA expression of 63 melanoma cancer cell lines obtained from the Cancer Cell Line Encyclopedia (CCLE; https://portals.broadinstitute.org/ccle) were analyzed.

*Patient cohort*

Formalin-fixed and paraffin-embedded benign (naevi) and primary malignant melanocytic lesions from 100 patients diagnosed between 2000 and 2008 were obtained from 4 French clinical centers: Centre Léon Bérard in Lyon, Hôpital de Bellevue in Saint Etienne, CHU Estaing in Clermont-Ferrand, Hôpital de la Timone in Marseille. The average age of the patients was 63.0 ± 16.5 years. 39% of the patients were men and 61% were women. The data concerning the histological type, the Clark level, the Breslow index, the development of metastases and the patient outcome were collected. Inclusion criteria were primary and metastatic melanoma previously diagnosed and with clinical follow-up of at least 5 years available. This work has been carried out in accordance with The Code of Ethics of the World Medical Association (Declaration of Helsinki) for experiments involving human samples. The research program, including studies on archival and stored materials, was approved by the research ethics committee of the Center Léon Bérard, Lyon, France.

*Detection of Tspan8 expression and presence of a BRAF^V600E^ mutation by immunohistochemistry*

Serial 4-µm paraffin-embedded primary melanoma sections were screened for Tspan8 protein expression as previously described (Berthier-Vergnes et al. Br J Cancer, 2011) with a mouse monoclonal antibody (TS29; 1/400; Le Naour et al, Mol Cell Proteomics, 2006) and for BRAFV600E mutation with a mouse monoclonal antibody (VE1ab228461, Abcam). Staining of eccrine glands in the dermis served as Tspan8 positive controls and tissue sections were scored positive if any reactive areas were seen in lesions. An immunointensity score (0: negative, 1: low, 2: moderate, 3: mild or 4: strong) was assigned to each sample after the scoring by two independent investigators including one in blind to avoid potential bias. Figure 3D illustrates for immunointensity scores of melanoma and in naevi samples (score 0) which have served as negative controls.

*Statistical analysis*

Statistical analyses were carried out with R software (version 3.4.3) (http://www. R-project.org/; Team 2008). All statistical tests were two tailed. Gaussian finite mixture models were applied using mclust R package (Scrucca et al, R J, 2016), and survival analyses were performed through survminer and survival R packages (Kosinski, <https://CRAN.R-project.org/package=survminer2018> ; Grambsch Springer, New York; 2000).
